# Double-Responsive Macrophage-Derived Exosomes Alleviate Acute Lung Injury

**DOI:** 10.34133/bmr.0277

**Published:** 2025-11-24

**Authors:** Chunhua Ma, Zhaocong Yang, Jing Wang, Xuemei Li, Tao Li, Liangming Liu

**Affiliations:** ^1^ Women and Children’s Hospital of Chongqing Medical University, Chongqing, China.; ^2^ Children’s Hospital of Nanjing Medical University, Nanjing 210008, China.; ^3^ School of Biology and Food Engineering, Institute of Pharmaceutical Pharmacology Research Center, Suzhou University, Suzhou, Anhui, China.; ^4^ State Key Laboratory of Trauma, Burns and Combined Injury, Shock and Transfusion Research Department of Army Medical Center, Army Medical University, Chongqing 400042, China.

## Abstract

Acute lung injury (ALI) is one of the complications of sepsis, and macrophages play an important role in ALI. The aim of this research was to investigate the effects of epidermal growth factor receptor (EGFR) monoclonal antibody-modified chemokine (C-X-C motif) ligand 8 (CXCL8) overexpression of macrophage (CXCL8@M)-derived exosomes miR-126a-3p (EGFR@CXCL8@exo-miR-126a-3p) on sepsis ALI. CXCL8@M was obtained via macrophage infection of CXCL8 plasmid, and CXCL8-M-exo was obtained via an exosome extraction kit. In addition, hsa-miR-126-3p agomir [a specially chemically modified microRNA (miRNA) mimic, named miR-126-3p] was loaded in CXCL8@M-exo to form CXCR8@exo-miR-126a-3p via electroporation technology. Further, EGFR@CXCR8@exo-miR-126a-3p was obtained via EGFR monoclonal antibody-modified CXCR8@exo-miR-126a-3p. Lipopolysaccharide (LPS)-induced ALI models were used to evaluate the role and mechanism of EGFR@CXCR8@exo-miR-126a-3p on ALI. Single-cell sequencing and miRNA chip results showed that miR-126a-3p was mainly expressed in pulmonary macrophages and markedly decreased, while single-cell sequencing and immunofluorescence results showed that EGFR was expressed and significantly elevated in macrophages in ALI mice. miR-126a-3p and EGFR siRNA significantly inhibited polarization of M1 macrophage. The imaging results of small animals showed that EGFR@CXCL8-exo-miR-126a-3p has obvious macrophage targeting. The results showed that EGFR@CXCR8@exo-miR-126a-3p significantly inhibited M1 macrophage and increased Treg cells to exert anti-inflammatory effects. The mechanism of EGFR@CXCR8@exo-miR-126a-3p on ALI is mainly via inhibition of PIK3R2/NLRP3 signaling pathway and ferroptosis. This study provided a new treatment method for ALI.

## Introduction

Sepsis is an organ dysfunction caused by a dysregulated body response caused by infection [1]. It is characterized by systemic immune system dysfunction, multiple organ dysfunction, and even death [2,3]. Epidemiological investigations have shown that the lung tissues were the target organs more susceptible to sepsis, with approximately half of sepsis patients experiencing acute lung injury (ALI) and a mortality rate exceeding 50% [4,5]. The mechanism of ALI in sepsis was currently unclear and may be the result of multiple mechanisms. The in-depth study of the molecular mechanisms of ALI was beneficial for the discovery of new therapeutic targets and has important value in improving the prognosis of ALI [6].

Multiple studies have found that microRNAs (miRNAs) regulated numerous posttranscriptional pathways involved in inflammatory responses [7–12] and are involved in the regulation of pyroptosis and apoptosis in lung [13–15]. Studies have shown that miR-126a-3p was significantly down-regulated in ALI. Serum miR-126-3p level was down-regulated in sepsis patients, and the degree of down-regulation was correlated with the severity of sepsis [16,17]. However, it is unclear whether miR-126-3p is involved in ALI.

Exosomes have unique morphological and compositional characteristics. They are contained in multivesicular bodies formed by the fusion of multiple endosomes and plasma membranes, with a diameter of 30 to 150 nm. They are vesicles with lipid bilayer membrane structure. Exosomes carry the characteristic biological information molecules of mother cells, such as protein, lipid, DNA, and noncoding RNA, which have natural molecular transport characteristics and good biocompatibility.

In sepsis- or LPS-induced ALI models, the epidermal growth factor receptor (EGFR) signaling pathway was activated in macrophages, which lead to macrophage activation and the secretion of pro-inflammatory cytokines [interleukin-1β (IL-1β) and IL-6] [18]. As a membrane receptor, EGFR signaling can provide a direct contact point for pathogens and enter host cells, and plays a crucial role in macrophages [19]. In addition, the EGFR inhibitor erlotinib inhibited excessive activation of macrophages and reduced the secretion of pro-inflammatory cytokines and protected sepsis lung injury [20]. The above indicated that EGFR could serve as one of the targeted macrophage indicator markers.

CXC chemokine ligand 8 (CXCL8), also known as IL-8, is secreted by monocytes, alveolar macrophages, endothelial cells, and epithelial cells [21]. In recent years, many researchers have devoted themselves to exploring the immunomodulatory effects of CXCL8. A previous study has found that the CXCL8/CXC chemokine receptor 2 (CXCR2) axis participates in mediating the transport and infiltration of M2 macrophages in pancreatic cancer [22]. In tissue samples with high expression of CXCL8, there is more infiltration of M2 macrophages, suggesting that M2 macrophage infiltration may be associated with high expression of CXCL8 [23]. Finally, there has been previous report of the presence of CXCL8 receptor CXCR1 on macrophages [24]. The above prompts indicated that CXCL8 was one of the important markers for targeting macrophages. Therefore, this study constructed engineered macrophage-derived exosomes that have EGFR and CXCL8 dual responses to deliver miR-126-3p for the treatment of ALI and also explored its mechanism.

## Materials and Methods

### Reagent

Lipopolysaccharide (LPS) was sourced from *Escherichia coli* 0111: B4, L2630 and purchased from Sigma-Aldrich (USA). hsa-miR-126-3p agomir (#HY-R00172A, a specially chemically modified miRNA mimic) was purchased from MedChemExpress Co. Ltd. Hematoxylin and eosin (H&E) staining kit was purchased from Beijing Soleibao Technology Co. Ltd. miR-126-3p primers (forward, 5′-CATACCTTGCGGTCAAACCAG-3′; reverse, 5′-TAGTCTCCTTGACACTCTCTC-3′), EGFR primers (forward, 5′-AGGCACGAGTAACAAGCTCAC-3′; reverse, 5′-ATGAGGACATAACCAGCCACC-3′), CXCL8 primers (forward, 5′-ACTGAGAGTGATTGAGAGTGGAC-3′; reverse, 5′-AACCCTCTGCACCCAGTTTC-3′), and PIK3R2 primers (forward, C 5′-CAGCAGTACCAGGACAAGA-3′; reverse, 5′-GCCTCAATTGCAGTACGCTT-3′) were purchased from GeneChem. PIK3R2 small interfering RNA (siRNA) (#SR419533) was purchased from ChemicalBook. PIK3R2 plasmid (NM_005027.4/5296) was purchased from Hanheng Biotechnology (Shanghai) Co. Ltd. EGFR monoclonal antibody (#4267), CXCL8 monoclonal antibody (#99407), NLRP3 monoclonal antibody (#15101), ASC monoclonal antibody (#13833), GSDMD-N monoclonal antibody (#39754), cleaved caspase-1 monoclonal antibody (#89332), caspase-1 monoclonal antibody (#2225), cleaved IL-1β monoclonal antibody (#63124), IL-1β monoclonal antibody (#12507), SLC7A11 monoclonal antibody (#98051), GPX4 monoclonal antibody (#52455), NRF2 monoclonal antibody (#20733), and GAPDH monoclonal antibody (#2188) were purchased from Cell Signaling Technology Company. PIK3R2 monoclonal antibody (#83606-1-PBS) was purchased from Proteintech. RAW264.7 cells were purchased from Beyotime Biotechnology.

### Animals

BALB/c [half male and half female, 6 weeks, 20 to 22 g, SYXK(hu)2023-0023] were obtained from the Shanghai SLAC Laboratory Animal Co. Ltd. Mouse feeding conditions: temperature of 20 to 25 °C, relative humidity of 45% to 65%, automatic darkroom for 12 h, no restriction on food and water intake, adaptive feeding for 1 week before starting the experiment. Bitter acid (3% to 5% yellow) or neutral red (0.5% red) was applied with a brush to different parts of the animal, and the numbers represented by each part are used to distinguish each mouse. All animals’ operations were approved by the Research Council and Animal Care and Use Committee of Nanjing Medical University (IACUC-2507088).

### Overexpressed CXCL8 macrophage (CXCL8@M) was established and identified

Macrophages were seeded in a 6-well plate. When the cell density was about 50% to 60%, fresh culture medium containing CXCL8 plasmid virus solution was added after cell exchange to maintain cell growth. Cell fluorescence was observed at 72 h, and the cells were cultured with puromycin for 1 week. The CXCL8@M cells were screened, cells with high purity and infection efficiency were obtained, and the CXCL8@M cells were established and identified via polymerase chain reaction (PCR) and Western blot.

### Overexpression of PIK3R2 in macrophages by PIK3R2 plasmids

Macrophages were seeded in a 6-well plate. When the cell density was about 50% to 60%, fresh culture medium containing PIK3R2 plasmid virus solution was added after cell exchange to maintain cell growth. Cell fluorescence was observed at 72 h, and the cells were cultured with puromycin for 1 week. The PIK3R2@M cells were screened, cells with high purity and infection efficiency were obtained, and the PIK3R2@M cells were established and identified via PCR (PIK3R2; forward primer, 5′-CCAGCAGTACCAGGACAAGA-3′; reverse primer, 5′-GCCTCAATTGCAGTACGCTT-3′) and Western blot. Macrophage cells were randomly divided into 4 groups: control group, LPS (1 μg/ml) group, LPS + CEm-126a-3p (20 μg/ml) + over PIK3R2 group. Macrophage cells were treated with ECEm-126a-3p (20 mg/ml) for 24 h, and 1 μg/ml LPS was co-incubated for 24 h, while the control group was challenged with equal phosphate-buffered saline (PBS).

### Silence PIK3R2 in macrophages

Macrophages were seeded in a 6-well plate. When the cell density was about 50% to 60%, fresh culture medium containing PIK3R2 siRNA solution was added after cell exchange to maintain cell growth. Cell fluorescence was observed at 72 h, and the cells were cultured with puromycin for 1 week. The PIK3R2 siRNA@M cells were screened, cells with high purity and infection efficiency were obtained, and the PIK3R2 siRNA@M cells were established and identified via PCR and Western blot. Macrophage cells were randomly divided into 4 groups: control group, LPS (1 μg/ml) group, LPS + CEm-126a-3p (20 μg/ml) + over PIK3R2 group. Macrophage cells were treated with ECEm-126a-3p (20 mg/ml) for 24 h, and 1 μg/ml LPS was co-incubated for 24 h, while the control group was challenged with equal PBS.

### qPCR detection of miR-126a-3p expression in exosomes between control and ALI mice

The expression level of miR-126a-3p in exo was detected by quantitative PCR (qPCR) technology, and the detection process was strictly carried out according to the instructions.

### Extraction of CXCL8@M-derived exosomes and preparation of CXCL8@exo-miR-126a-3p

The CXCL8@M cells were taken from a −80 °C freezer, thawed, and placed on an ice box for use. They were centrifuged at 3,000*g* at 4 °C for 10 min, and the upper layer of liquid was aspirated and centrifuged at 10,000*g* in a 4 °C centrifuge for 20 min. The supernatant was aspirated and transferred to a new centrifuge tube. The steps for separating extracellular vesicles were strictly carried out according to the instructions. The centrifuge tube was placed in a precooled centrifuge at 4 °C and centrifuged at 12,000*g* to obtain a purified CXCL8@exo solution. The purified exosomes were observed for exo morphology by transmission electron microscopy, and the range of exo particle size was determined by nanoparticle tracking analysis. Immunoblotting was used to detect the expression of typical markers CXCL8, CD9, CD63, CD81, TSG101, and calnexin. miR-126a-3p inhibitor was introduced into CXCL8@exo through electroporation technology (the electrical conversion conditions are a voltage of 30 V and 6 pulses) to obtain CXCL8@exo-miR-126a-3p.

### EGFR monoclonal antibody-modified CXCL8@exo-miR-126a-3p

In total, 1.5 times the amount of catalyst [1-ethyl-3- (3-dimethylaminopropyl) carbodiimide] (EDC) and *n*-hydroxysuccinimide (NHS) were added to PD-1-M-M, centrifuged at low-temperature overspeed (20,000 rpm × 20 min), and resuspended in 10 ml of PBS with pH 6.5. An appropriate amount of EGFR monoclonal antibody was added for 4 h. EGFR monoclonal antibody molecules, EDC, and NHS reagents that were not coupled to CXCL8@exo-miR-126a-3p were separated multiple times by low-temperature ultracentrifugation with deionized water at pH 7.4. The precipitate was resuspended in PBS to obtain EGFR@CXCL8@exo-miR-126a-3p.

### The effects of EGFR@CXCL8@exo-miR-126a-3p on ALI

The mice were randomly divided into the following groups: control group, LPS (10 mg/kg, intraperitoneal injection), LPS + EGFR@CXCL8@exo-miR-126a-3p (ECEm-126a-3p, 100 μg/kg, intraperitoneally), and LPS + CXCL8@exo-miR-126a-3p (CEm-126a-3p, 100 μg/kg, intraperitoneally). After 24 h of intervention of LPS, EGFR@CXCL8@exo-miR-126a-3p and CXCL8@exo-miR-126a-3p (caudal vein injection) were given to mice for 3 consecutive days.

Macrophage were randomly divided into 4 groups: control, LPS (1 μg/ml), LPS + ECEm-126a-3p (20 μg/ml), and LPS + CEm-126a-3p (20 μg/ml). M2 cells were treated with ECEm-126a-3p (20 mg/ml) and CEm-126a-3p (20 mg/ml) for 24 h, and 1 μg/ml LPS was co-incubated for 24 h, while the control group was challenged with an equal PBS.

### Lung dry wet specific gravity detection

The lung tissues of mice were separated, the left and right lung tissues were cut open, the left lung tissues were removed and washed with PBS, and the surface moisture was absorbed, weighed, and baked in an 80 °C oven for 36 h. The dry weight was measured, and lung dry wet specific gravity (W/D) was calculated.

### Detection of lung function

After intraperitoneal injection of 0.4% pentobarbital sodium solution (0.2 ml/10g) into mice for anesthesia, they were placed in a supine position and fixed on a wooden board. After tracheal intubation, the mice were transferred to a dynamic monitoring box to monitor their respiratory status. The respiratory rate of the ventilator was set to 90 times/min, with a respiratory ratio of 20:10. After the system showed that the spontaneous respiration of the mice had disappeared and entered a stable stage, the minimum values of inspiratory airway resistance (RL) and expiratory airway resistance (Re), the average value of lung dynamic compliance (Cdyn), and the average value of maximum ventilation volume per minute (MVV) were recorded within 3 min. After the experiment was completed, data collection was stopped and saved.

### Pathological changes in lung tissues

The right lung tissues of mice in 4% paraformaldehyde were fixed for 48 h, and H&E staining was used to evaluate the pathological changes in the lung tissues of mice. The pathological scores of lung tissues were performed via double-blind experiment, and the scores were evaluated according to the following content according to previous report [25].

### Lung targeting of EGFR@CXCL8@exo-miR-126a-3p

#### DiR-labeled EGFR@CXCL8@exo-miR-126a-3p

Fluorescent lipophilic tracer DiR (1 mM) was added into EGFR@CXCL8@exo-miR-126a-3p for 15 min at 25 °C, and DiR- EGFR@CXCL8@exo-miR-126a-3p was obtained.

#### Animal imaging analysis

Cyanine 5.5 (Cyc5.5)-EGFR@CXCL8@exo-miR-126a-3p (60 μl) was injected into the mice via tail vein. The mice were placed in an animal live imaging device for live imaging.

### Validation of miR-126a-3p target genes

#### Screening of miR-126a-3p target genes

The bioinformatics software TargetScan (https://www.targetscan.org/vert_80/) and miRDB (https://www.miRDB.org) database predicted the target genes of miR-126a-3p.

#### Dual luciferase experiment

The target fragment was inserted into the reporter gene vector expressing luciferase. Experimental groups: PIK3R2-3′UTR (untranslated region) (wild type) + miR-NC, PIK3R2-3′UTR (wild type) + miR-126a-3p, PIK3R2-3′UTR (mutant) + miR-NC, PIK3R2-3′UTR (mutant) + miR-126a-3p. For double-luciferase activity experiment, the operation process of the dual luciferase experiment strictly follows the instructions.

### Single-cell sequencing of the lung tissues

After obtaining the lung of each group of mice, a single-cell suspension with a concentration of 118 cells per milliliter was prepared. The 10× Genomics Chromium system was used to free mRNA from the single-cell microreactor system Gel Beam in Impulse (GEMs), and mRNA was reverse transcribed into cDNA. After amplification, purification, and enrichment, the qualified cDNA was constructed into a 3′ transcriptome library. The NovaSeq XPlus second-generation sequencing platform was used via PE150 sequencing mode for sequencing.

### Western blot

The lower lobe of the right lung was added to radioimmunoprecipitation assay (RIPA) lysis buffer in a weight to volume ratio (1:10), ground with steel balls, and placed on ice for 20 min to fully lyse the protein. After centrifugation, the supernatant was taken for ultrasonic lysis. The Bicinchoninic Acid Protein Assay Kit method was used to determine the total protein concentration. All samples were electrophoretized on sodium dodecyl sulfate–polyacrylamide gel electrophoresis (SDS-PAGE) gel and transferred to polyvinylidene difluoride (PVDF) membrane. The primary antibodies were incubated overnight after being blocked with rapid blocking solution. The membrane was washed; the secondary antibody was incubated at room temperature for 1 h. The enhanced chemiluminescence method was used for developing strips, and the grayscale values of the bands were analyzed using ImageJ image analysis software.

### Immunohistochemistry and immunofluorescence

The thickness of the fixed and embedded lung tissues slices was 3 μm, and immunostaining was performed using specific antibodies against the relevant proteins. Slices were immunostained using diluted rabbit anti-related polyclonal antibodies. The primary antibody was incubated overnight at 4 °C, and the following day, the secondary antibody was incubated at room temperature for 2 h. Slices were stained with 4′,6-diamidino-2-phenylindole (DAPI) at room temperature for 10 min and scanned using a tissue section digital scanner.

### Statistical analysis

All data were expressed as mean ± standard deviation (SD) and analyzed by one-way analysis of variance (ANOVA) followed by Tukey multiple comparison test using GraphPad Prism 8 (GraphPad Software, USA). A value of *P* < 0.05 was considered statistically significant.

## Results

### EGFR was significantly increased in lung tissues of ALI mice

**Fig. 1.**
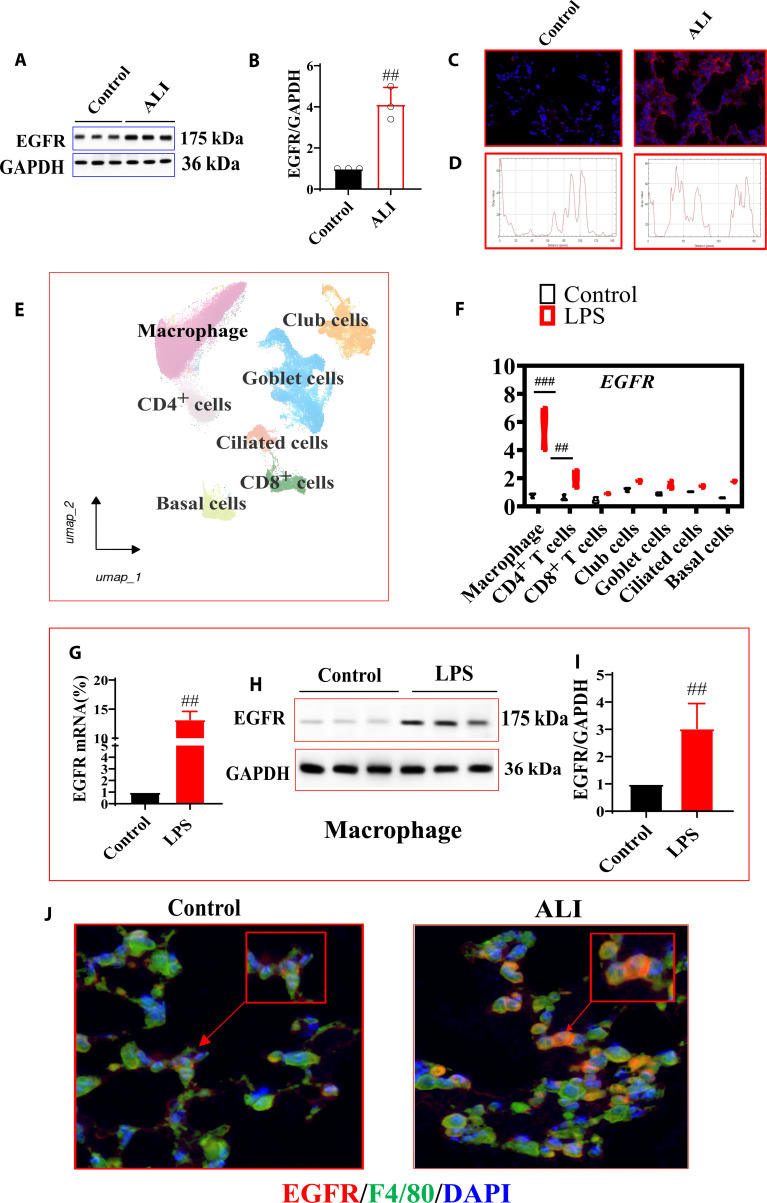
Epidermal growth factor receptor (EGFR) was significantly increased in acute lung injury (ALI). (A and B) Protein level of EGFR in lung tissues of ALI mice (*n* = 3). (C and D) The protein level of EGFR was detected through immunofluorescence technology (*n* = 3). (E and F) Single-cell sequencing detection of EGFR expression in different cells of lung tissues (*n* = 3). (G) mRNA level of EGFR in LPS-induced M cells (*n* = 6). (H and I) Protein level of EGFR in LPS-induced M cells (*n* = 3). (J) Colocalization of EGFR and macrophages (*n* = 3). All data were presented as mean ± SD. Compared with control group: ^#^*P* < 0.05, ^##^*P* < 0.01.

A previous study has shown that EGFR plays an important role in ALI. Therefore, the level of EGFR in lung tissues was detected in this study. The results showed that the level of EGFR was significantly increased in LPS mice as compared with control mice (Fig. 1A to D). Due to the presence of multiple cells in lung tissues, which type of cell primarily expresses EGFR was the key determining factor in the study. Single-cell RNA sequencing (RNA-seq) was used between ALI mice and control mice. The results showed that mRNA of EGFR was up-regulated in macrophages in lung tissues of ALI compared with lung tissues of control mice (Fig. 1E to G).

To further verify the expression of EGFR in macrophages, qPCR and Western blot were used to detect the mRNA and protein levels of EGFR. The results showed that the mRNA and protein levels of EGFR were significantly decreased as compared with the control group (Fig. 1H to J). Further, detection of colocalization between EGFR and macrophages (macrophage marker F4/80) was via immune colocalization. The results showed that EGFR exhibited significant colocalization with macrophages (Fig. 1K).

### EGFR regulated polarization of macrophage

**Fig. 2.**
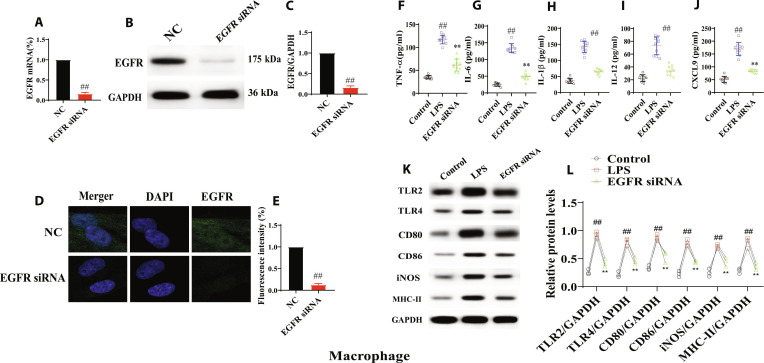
EGFR regulated macrophage polarization in ALI. (A to C) mRNA and protein level of EGFR in EGFR siRNA-M cells (*n* = 6). (D and E) mRNA and protein level of EGFR in EGFR siRNA-M cells via immunofluorescence technology (*n* = 3). (F to J) Levels of IL-6, IL-1β, TNF-α, IL-12, and CXCL9 in EGFR siRNA-M cells (*n* = 6). (K and L) Levels of TLR2, TLR4, CD80, CD86, iNOS, and MHC-II in EGFR siRNA-M cells. All data were presented as mean ± SD. Compared with control group: ^#^*P* < 0.05, ^##^*P* < 0.01.

RAW264.7 cells were used to evaluate the effect of EGFR on macrophage polarization. RAW264.7 cells (M0) polarized into M1 macrophages under LPS (100 ng/ml) stimulation, and the M1 macrophage-related cytokines [tumor necrosis factor-α (TNF-α), IL-1β, IL-6, IL-12, and CXCL9] and surface markers [Toll-like receptor 2 (TLR2), TLR4, CD80, CD86, inducible nitric oxide synthase (iNOS), and major histocompatibility complex class II (MHC-II)] were detected. Firstly, EGFR specifically knocked macrophages (EGFR siRNA) were established. Compared with the NC group, the mRNA and protein levels of EGFR were significantly decreased (Fig. 2A to E). Compared with M0, the levels of TNF-α, IL-1β, IL-6, IL-12, and CXCL9, as well as those of TLR2, TLR4, CD80, CD86, iNOS, and MHC-II, were increased. Knockout EGFR M1 (EGFR siRNA) macrophages were built and restored those changes (Fig. 2F to L).

### Differential miRNAs in ALI mice and qPCR detection

**Fig. 3.**
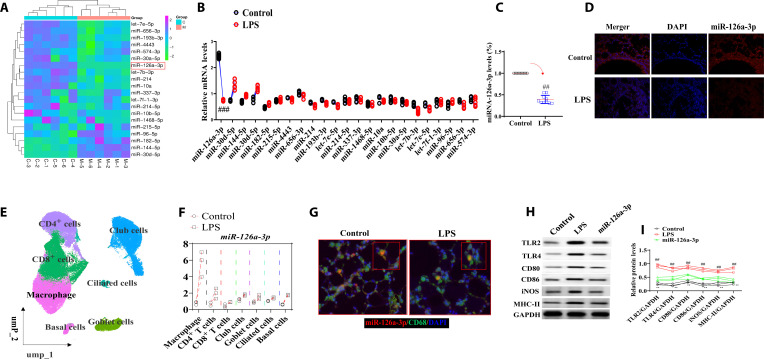
miR-126a-3p was significantly decreased and regulated macrophage polarization in ALI. (A to C) Detection of differentially expressed miRNAs in ALI using mRNA chips (*n* = 6). (D) MicroRNA scope staining of miR-126a-3p in lung tissues (×10, *n* = 3). (E and F) Single-cell sequencing of miR-126a-3p in lung tissues (*n* = 3). (G) Colocalization of miR-126a-3p and macrophages (*n* = 3). (H and I) Levels of TLR2, TLR4, CD80, CD86, iNOS, and MHC-II in over miR-126a-3p M cells. All data were presented as mean ± SD. Compared with control group: ^#^*P* < 0.05, ^##^*P* < 0.01.

In this study, the expressions of multiple miRNAs were detected via RNA chips. Twenty-two differentially expressed miRNAs in lung were detected. Among them, miR-126a-3p was significantly down-regulated most obviously (Fig. 3A to C). Therefore, miR-126a-3p was selected for our research. To further confirm the level of miR-126a-3p in ALI, the level of miR-126a-3p was detected by microRNA Scope staining (red probe) and qPCR. The results of the RNA Scope staining showed that compared with control group, the level of miR-126a-3p was decreased in lung tissues of ALI mice (Fig. 3D). The differential expression ofmiRNA126a-3p on different cells in lung tissue were detected. Single-cell RNA-seq was used between ALI mice and control mice. The result showed that miR-126a-3p was mainly expressed in macrophages (Fig. 3E and F). Next, we detected the level of miRNA126a-3p on macrophages. This study conducted costaining of microRNA Scope staining and macrophages (CD68, green). As we expected, the level of miR-126a-3p was decreased in CD68^+^ cells (Fig. 3G).

### miR-126a-3p regulated the polarization of macrophage

RAW264.7 cells (M0) polarized into M1 macrophages under LPS (100 ng/ml) stimulation and the M1 macrophage-related cytokines (TNF-α, IL-1β, and IL-6) and surface markers (TLR2, TLR4, CD80, CD86, iNOS, and MHC-II) were detected. Compared with M0, the levels TLR2, TLR4, CD80, CD86, iNOS, and MHC-II were increased. miR-126a-3p siRNA M1 macrophages (miR-126a-3p siRNA) were built and reduced those changes of the above indicators (Fig. 3H and I).

### Construction and characterization of EGFR@CXCR8@exo-miR-126a-3p

In order to construct engineered exosomes targeting macrophages, macrophage-derived exosomes (exo) were used in this study. Chemokine CXCL8 is a cytokine secreted by macrophages and epithelial cells. CXCL8 could bind to CXCL8 receptor on the macrophage membrane. Therefore, constructed engineered exosomes (CXCL8-exo) could target and recognize the CXCR1 receptor, thereby achieving the goal of targeting macrophages. Firstly, overexpressed CXCL8 lentivirus was transfected to the macrophage. The CXCL8 mRNAs and protein levels were detected. The results showed that the mRNA and protein levels of CXCL8 were increased as compared with the vector group. CXCL8-exo was obtained from CXCL8@M in this study (Fig. S1A to C). Similarly, the CXCL8 mRNAs and protein levels were significantly increased in CXCL8@exo as compared with exo (Fig. S1D to F).

**Fig. 4.**
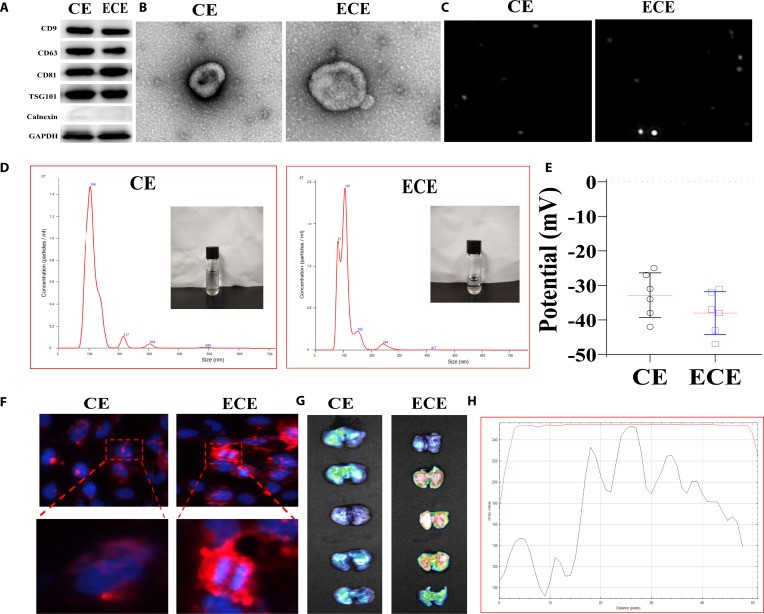
Construction and identification of EGFR@CXCL8-exo-miR-126a-3p. (A) Detection of exosome protein markers such as CD9, CD63, CD81, TSG101, and calnexin. (B) Transmission electron microscopy of EGFR@CXCL8-exo-miR-126a-3p and CXCL8-exo-miR-126a-3p. (C to E) Particle size analysis of EGFR@CXCL8-exo-miR-126a-3p and CXCL8-exo-miR-126a-3p. (F) Uptake of EGFR@CXCL8-exo-miR-126a-3p and CXCL8-exo-miR-126a-3p in M cells. (G and H) Imaging results of small animals.

Western blot was used to detect the expression of CD9, CD63, CD81, TSG101, and calnexin-related indicators for CXCL8-exo and EGFR@ CXCL8@exo. CD9, CD63, CD81, and TSG101 were highly expressed in CXCL8-exo and EGFR@CXCL8@exo, while calnexin was not expressed (Fig. 4A). Under transmission electron microscopy, CXCL8-exo (CE) and EGFR@CXCL8@exo (ECE) exhibited a cup-shaped lipid bilayer structure with distinct boundaries (Fig. 4B). The nanoparticle tracking analysis (NTA) results showed that the size range of CXCL8@exo and EGFR@CXCL8@exo was 90 to 200 nm (Fig. 4C to E).

EGFR@CXCL8@exo-miR-126a-3p was established by importing miR-126a-3p mimic using the electroporation technology. Then, the take-up of EGFR@CXCL8@exo-miR-126a-3p by M1 was measured. More EGFR@CXCL8@exo-miR-126a-3p were taken up than CXCL8@exo-miR-126a-3p (Fig. 4C to E). 3,3′-Dioctadecyloxacarbocyanine perchlorates (Dio)@EGFR@CXCL8@exo-miR-126a-3p and Dio@CXCL8@exo-miR-126a-3p were injected into mice through the tail vein. The amount of (Dio)@EGFR@CXCL8@exo-miR-126a-3p was significantly increased than Dio@CXCL8@exo-miR-126a-3p in lung tissues (Fig. 4F and G).

### Prediction analysis of miR-126a-3p target gene-related signaling pathways

**Fig. 5.**
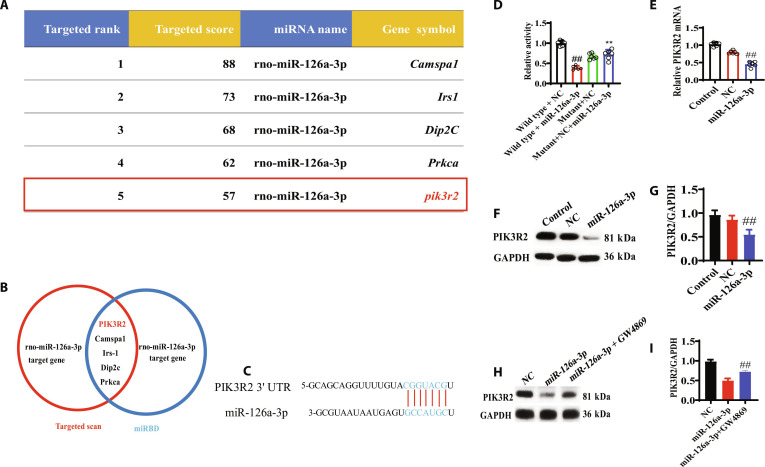
Search for miR-126a-3p target gene. (A) Prediction of miR-126a-3p target genes in the miRDB database. (B) Intersection between TargetScan and miRDB database miR-126a-3p target genes. (C) TargetScan software predicts the binding relationship between PIK3R2 and miR-126a-3p. (D) Detection of targeted binding between miR-126a-3p and PIK3R2 using dual luciferase assay. (E) Real-time PCR detection of PIK3R2 mRNA expression in M cells in each group. (F and G) Western blot detection of PIK3R2 protein expression in M cells of each group. (H and I) Changes in PIK3R2 protein expression in M cells of each group after GW4869 intervention. All data were presented as mean ± SD. Compared with NC group: ^#^*P* < 0.05, ^##^*P* < 0.01.

Five candidate genes (*PIK3R2*, *Camsap1*, *Irs1*, *Dip2c*, and *Prkca*) were selected as the intersection target genes. PIK3R2 was closely related to sepsis and regulated pathological processes in the mechanism of sepsis. Therefore, PIK3R2 was ultimately selected as the target gene for miR-126a-3p for subsequent research (Fig. 5A to C).

Compared with the PIK3R2-3′UTR (wild type) + miR-NC group, the PIK3R2-3′UTR (wild type) + miR-126a-3p group showed a significant decrease in luciferase activity fold values, while the PIK3R2-3′UTR (mutant) + miR-NC group and the PIK3R2-3′UTR (mutant) + miR-126a-3p group showed no significant difference. The above results indicated the targeted binding between miR-126a-3p and PIK3R2 (Fig. 5D to G).

The results showed that compared with the control group, the mRNA level of PIK3R2 in the exo-miR-126a-3p group was significantly down-regulated. GW4869 (an inhibitor of exosome synthesis/release) was used to treat cells in the miR-126a-3p group. The results showed that compared with the NC group, the level of PIK3R2 in the miR-126a-3p group was significantly down-regulated. Compared with the miR-126a-3p group, the miR-126a-3p + GW4869 group showed a significant up-regulation of PIK3R2 protein levels, further confirming the targeting relationship between miR-126a-3p and PIK3R2 (Fig. 5H and I).

**Fig. 6.**
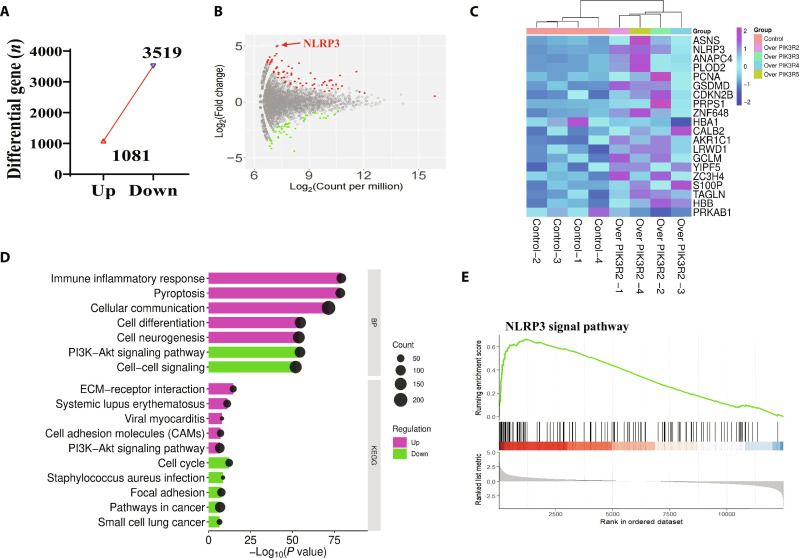
Search for PIK3R2 target gene in overexpressed M cells. (A) Number of up-regulated and down-regulated genes (*n* = 6). (B) Differential gene volcano map (*n* = 6). (C) Top 20 gene enrichment rankings. (D) Enrichment of signaling pathways (*n* = 6). (E) NLRP3 signaling pathway (*n* = 6).

In order to identify the downstream function of PIK3R2 in macrophage, RNA was extracted for transcriptome sequencing. The results showed that compared with the control group, the overexpressed *PIK3R2* M showed a significant up-regulation of 1,081 genes and down-regulation of 3,519 genes (Fig. 6A and B). Through bioinformatics analysis, it was found that the *NLRP3* gene was up-regulated by more than 2-fold and was the most changed and aroused our research interest (Fig. 5B to E). Firstly, the expression of NLRP3 in macrophages was evaluated when treated with LPS in *PIK3R2* siRNA macrophages, the mRNA expression of NLRP3 in macrophages has significantly decreased, and the protein expression of NLRP3 in LPS-induced macrophages has also significantly decreased (Fig. S2A to C). The above results suggested that NLRP3 was one of the downstream signaling proteins of PIK3R2. In addition, compared with the LPS + exo-miR-126a-3p + vector, the LPS + exo-miR-126a-3p + overexpressed PIK3R2 group showed significant up-regulation of the levels of NLRP3, ASC, GSDMD-N, cleared IL-1β, and cleared caspase-1 (Fig. S2D and E).

On the other hand, in order to search for the direct signaling pathway of EGFR@CXCL8@exo-miR-126a-3p (ECEm-126-3p), RNA-seq analysis in lung tissues was used in this study. As shown in the Venn diagram, there are 652 genes shared among the 3 groups of differentially expressed genes (Fig. S3A). All shared differentially expressed genes (DEGs) were clustered in the heatmap, showing significant differences in mRNA levels between the control group (A) and the LPS group (B). Compared with the LPS group, mRNA in the ECEm-126-3p group (C) was recovering toward the control group. Enrichment through signaling pathways showed that the main differential signals were concentrated in the iron ion signaling pathway, with the most significant changes observed (Fig. S3B). The hotspot map results showed the top 20 genes with significant changes, and SLC7A11 was decreased in the LPS group and increased in the treatment group (Fig. S3C). The above results suggested that the iron ion signaling pathway may be the main signaling pathways through which ECEm-126-3p exerts its activity.

### Macrophage-regulated Treg cell function

The levels of regulatory T (Treg) cells (Tregs) in the peripheral blood of sepsis patients were decreased. A recent study has shown that macrophages affected the proliferation, migration, and function of Tregs through various pathways. Macrophages expressed CCL1, which was the ligand for CCR8 and was expressed on Tregs, attracted Tregs into the target area. Secondly, macrophages secreted CCL22 to induce Treg cell migration to the target area. Therefore, in this study, tracheal injection of macrophage scavenger clodronate liposomes (Clod-Lip) was used to clear macrophages in lung tissues. After 24 h, immunofluorescence was used to confirm whether macrophages were successfully cleared. Compared with the control liposome (Con-Lip) group, the fluorescence intensity of F4/80 was significantly reduced in the Clod-Lip group. This result indicated that the macrophages in lung tissues have been cleared. After LPS was administrated to Con-Lip mice and Clod-Lip mice, compared with Con-Lip mice, Clod-Lip mice showed reduced Tregs in lung tissues. To further determine the regulatory role of macrophages on Tregs, this experiment constructed CCL1- or CCL22-specific knockout macrophage (CCL1 siRNA-M or CCL22 siRNA-M) to evaluate the migration capability of Tregs. Cell supernatant was collected after LPS-induced M cells (M-C) and CCL1 siRNA-M cells (CCL1 siRNA-M-C or CCL22 siRNA-M-C) and then M-C and CCL1 siRNA-M-C or CCL22 siRNA-M-C were used to cultivate Tregs, and cell scratch assay was used to investigate Treg cell migration ability. The results showed that M-C significantly increased the migration ability of Tregs as compared to the CCL1 siRNA-M-C and CCL22 siRNA-M-C groups. The above results suggested that M cells regulated Tregs through CCL1 and CCL22 (Fig. S4A to D).

### EGFR@CXCL8@exo-miR-126a-3p alleviated ALI in mice by regulating macrophage to control Tregs

LPS significantly reduced the survival rate of ALI mice. ECEm-126a-3p significantly increased the survival rate. Compared with the control group, the levels of W/D and myeloperoxidase (MPO) in the LPS group were significantly increased, while in the ECEm-126a-3p group, the levels of W/D and MPO were significantly decreased. Compared with the control group, the levels of IL-1β, IL-6, and TNF-α in serum, lung tissues, and bronchoalveolar lavage fluid (BLAF) of the LPS group were significantly increased, while ECEm-126a-3p significantly restored those changes (Figs. S5A to G and S6).

**Fig. 7.**
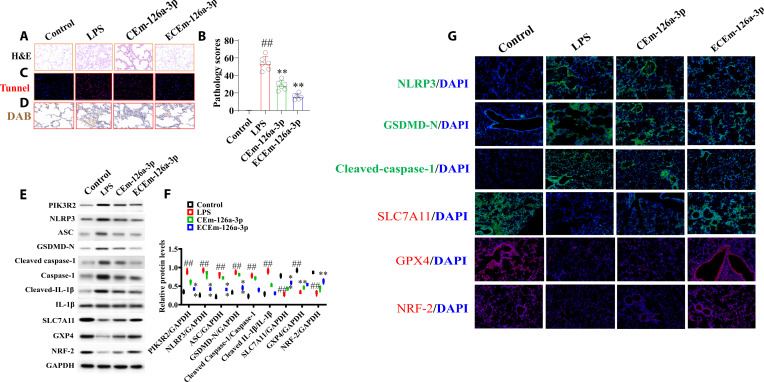
The effects of EGFR@CXCL8-exo-miR-126a-3p on ALI mice. (A and B) H&E staining of lung tissues (*n* = 6). Pathological scores of lung tissues (*n* = 6). (C) TUNEL (terminal deoxynucleotidyl transferase–mediated deoxyuridine triphosphate nick end labeling) staining of lung tissues (*n* = 6). (D) Diaminobenzidine (DAB) staining of lung tissues (*n* = 6). (E and F) Levels of PIK3R2, NLRP3, ASC, GSDMD-N, cleaved caspase-1, cleaved IL-1β, SLC7A11, GPX4, and NRF-2 of lung tissues (*n* = 6). (G) Levels of NLRP3, GSDMD-N, cleaved caspase-1, SLC7A11, GPX4, and NRF-2 of lung tissues (*n* = 6). All data were presented as mean ± SD. Compared with control group: ^#^*P* < 0.05, ^##^*P* < 0.01. Compared with LPS group: *P < 0.05, **P < 0.01.

After LPS infusion, the branching structures of various levels of bronchi in the lungs of mice were incomplete. The alveolar epithelial cells undergo vacuolar degeneration or necrosis, with uneven cytoplasmic staining or vacuolization, nuclear condensation or fragmentation, and a small number of cells shedding into the alveolar cavity. A small or large number of rod-shaped neutrophils and round deeply stained lymphocytes can be seen infiltrating the alveolar interstitial. Compared with the LPS group, the pathological changes of lung in the ECEm-126a-3p group were significantly decreased (Fig. 7A and B).

Compared with the control group, the levels of Fe^2+^, PIK3R2, NLRP3, ASC, GSDMD-N, cleaved caspase-1, and cleaved IL-1β significantly increased and SLC7A11, GPX4, and NRF-2 were significantly decreased in the LPS group, while in the ECEm-126a-3p group, the levels of PIK3R2, NLRP3, ASC, GSDMD-N, cleaved caspase-1, and cleaved IL-1β significantly decreased and SLC7A11, GPX4, and NRF-2 were significantly decreased (Fig. 7C to F).

Finally, the effects of ECEm-126a-3p on Tregs were evaluated to verify that ECEm-126a-3p regulated macrophage to control Tregs. The results of flow cytometry analysis showed that ECEm-126a-3p significantly increased the percentage of Tregs in lung tissues, and Western blot results showed that ECEm-126a-3p significantly increased the level of Foxp3 (Fig. S7A and B).

### EGFR@CXCL8@exo-miR-126a-3p regulated macrophage to control Tregs

Compared with the control group, the levels of IL-1β, IL-6, and TNF-α in the LPS group significantly increased, while in the ECEm-126a-3p group, the levels of IL-1β, IL-6, and TNF-α significantly decreased (Fig. S8A and B).

**Fig. 8.**
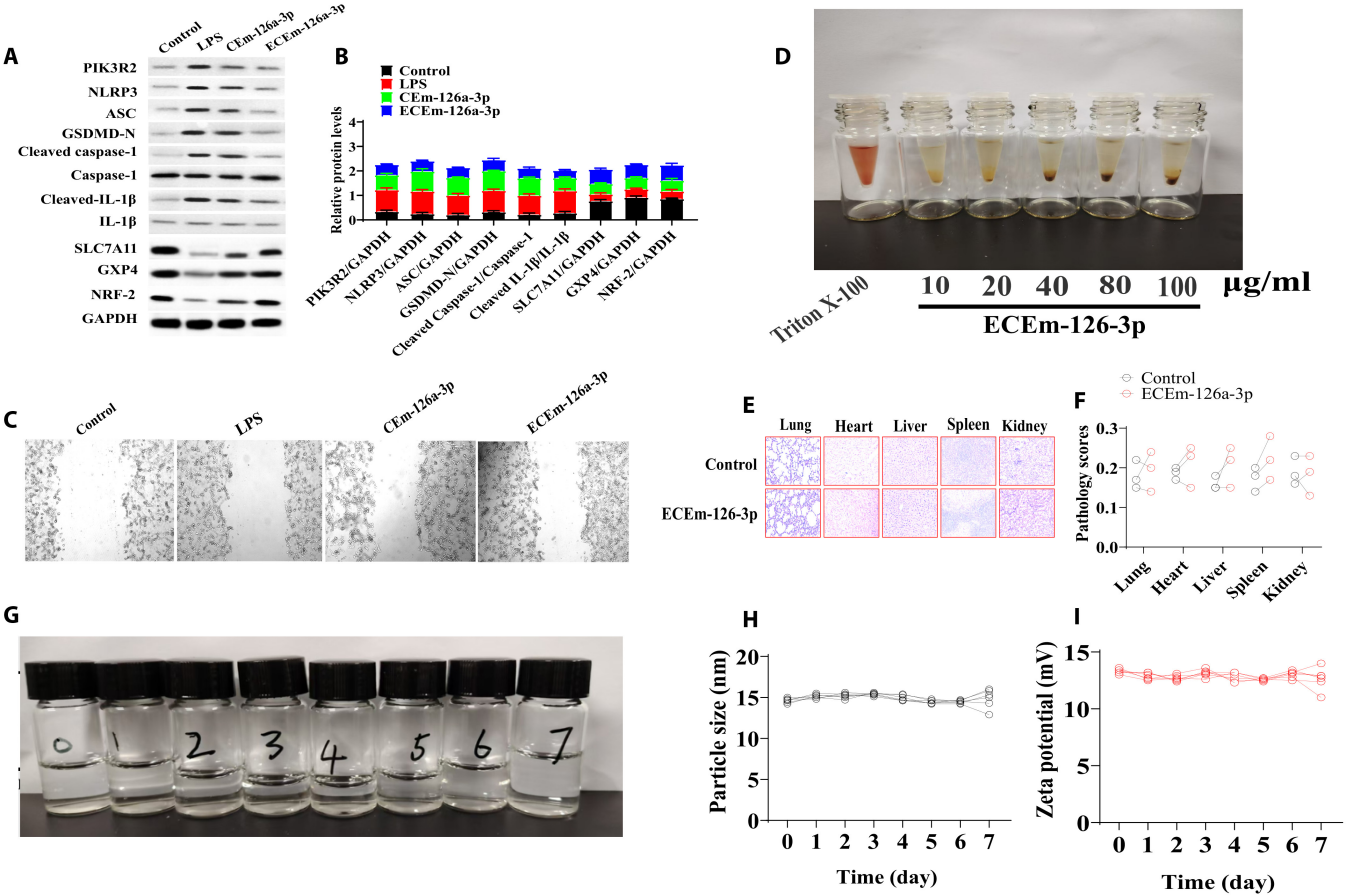
The effects of EGFR@CXCL8-exo-miR-126a-3p on LPS-induced M cells and safety evaluation of EGFR@CXCL8-exo-miR-126a-3p. (A and B) Levels of PIK3R2, NLRP3, ASC, GSDMD-N, cleaved caspase-1, cleaved IL-1β, SLC7A11, GPX4, and NRF-2 of lung tissues (*n* = 6). (C) EGFR@CXCL8-exo-miR-126a-3p-stimulated M cell supernatant to promote Treg cell migration (*n* = 6). (D) Hemolysis test (*n* = 6). (E and F) Effect of EGFR@CXCL8-exo-miR-126a-3p on the liver, liver, spleen, lungs, and kidneys of normal mice (*n* = 6). (G to I) Stability of EGFR@CXCL8-exo-miR-126a-3p within 7 d (*n* = 6). All data were presented as mean ± SD. Compared with control group: ^#^*P* < 0.05, ^##^*P* < 0.01. Compared with LPS group: *P < 0.05, **P < 0.01.

Compared with the control group, the levels of PIK3R2, NLRP3, ASC, GSDMD-N, cleaved caspase-1, and cleaved IL-1β significantly increased and SLC7A11, GPX4, and NRF-2 were significantly decreased in the LPS group, while in the ECEm-126a-3p group, the levels of PIK3R2, NLRP3, ASC, GSDMD-N, cleaved caspase-1, and cleaved IL-1β significantly decreased and SLC7A11, GPX4, and NRF-2 significantly increased. The effects of ECEm-126a-3p on Tregs were evaluated to verify that ECEm-126a-3p regulated macrophage to control Tregs in vitro*.* ECEm-126a-3p-stimulated macrophage supernatant (ECEm-126a-3p-C) was used to cultivate Tregs. The results of flow cytometry showed that ECEm-126a-3p-C significantly increased the percentage of Tregs in lung tissues (Fig. 8A to C).

### Safety evaluation of EGFR@CXCL8@exo-miR-126a-3p in vivo

The hemolysis rates of different concentrations of EGFR@CXCL8@exo-miR-126a-3p were all less than 5%, which meets the safety standards of the national standard for hemolysis rates and has no hemolytic properties (Fig. 8D). Compared with the control group mice, the heart, liver, spleen, lungs, and kidneys of EGFR@CXCL8@exo-miR-126a-3p group mice all maintained good integrity and no obvious injury (Fig. 8E). Compared with the results of the first 0 d, the particle size and electric potential of EGFR@CXCL8@exo-miR-126a-3p did not change significantly after being refrigerated at 4 °C for 7 d (Fig. 8F and G).

### The effects of PIK3R2 on macrophage

**Fig. 9.**
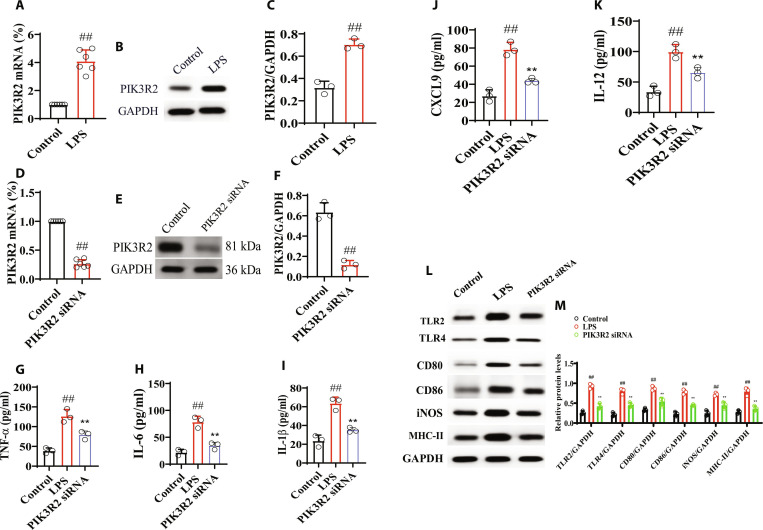
The effects of PIK3R2 on macrophage. (A) mRNA of PIK3R2 in LPS-induced macrophage (*n* = 3). (B and C) mRNA and protein expressions of PIK3R2 in LPS-induced macrophage (*n* = 3). (D to F) mRNA and protein expressions of PIK3R2 in PIK3R2-treated macrophage (*n* = 3). (G to K) Levels of TNF-α, IL-1β, IL-6, IL-12, and CXCL9 in PIK3R2-treated macrophage (*n* = 6). (L and M) Levels of TLR2, TLR4, CD80, CD86, iNOS, and MHC-II in PIK3R2-treated macrophage (*n* = 3). All data were presented as mean ± SD. Compared with control group: ^#^*P* < 0.05, ^##^*P* < 0.01. Compared with LPS or PIK3R2 siRNA group: *P < 0.05, **P < 0.01.

Firstly, we evaluated the expression of PIK3R2 in LPS-induced macrophage model. The results showed that the mRNA and protein expressions of PIK3R2 significantly increased in LPS-induced macrophage (Fig. 9A to C). In addition, the effect of PIK3R2 on macrophage polarization was evaluated. The results showed that PIK3R2 siRNA significantly decreased the levels of TNF-α, IL-1β, IL-6, IL-12, and CXCL9 and also the levels of TLR2, TLR4, CD80, CD86, iNOS, and MHC-II (Fig. 9E to J). The above results suggested that PIK3R2 could directly interact with macrophages.

### The effects of EGFR@CXCL8@exo-miR-126a-3p on NLRP3 signaling pathway and ferroptosis in LPS-induced macrophage model via PIK3R2

**Fig. 10.**
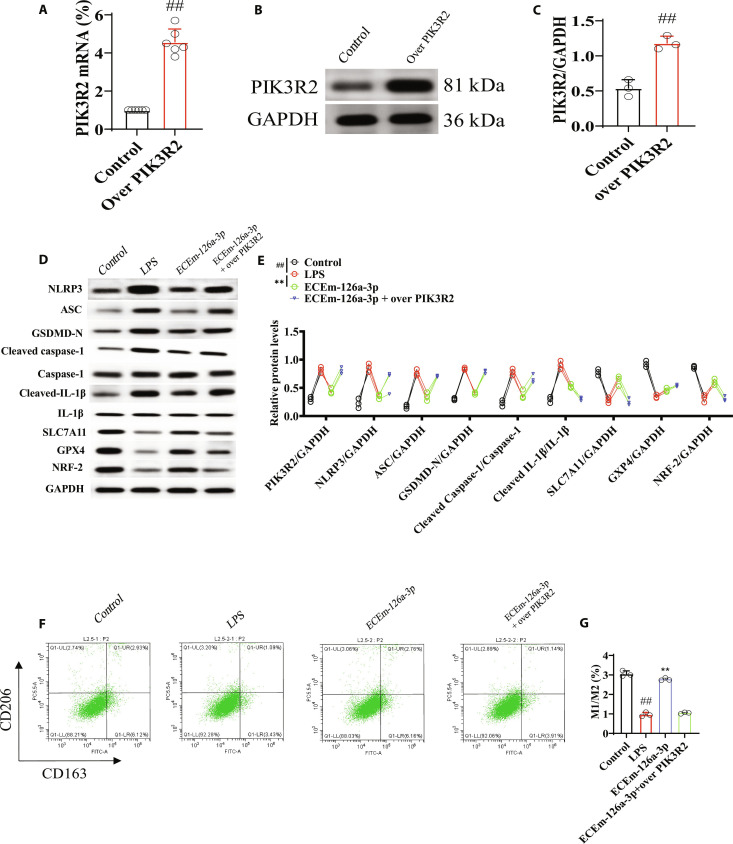
The effects of EGFR@CXCL8@exo-miR-126a-3p on NLRP3 signaling pathway and ferroptosis in LPS-induced macrophage model via PIK3R2. (A) mRNA of PIK3R2 in over PIK3R2 macrophage (*n* = 3). (B and C) Protein expressions of PIK3R2 in over PIK3R2 macrophage (*n* = 3). (D and E) Levels of NLRP3, ASC, GSDMD-N, cleaved caspase-1, and cleaved IL-1β and increased SLC7A11, GPX4, and NRF-2 in over PIK3R2 macrophage (*n* = 3). (F and G) Effects of EGFR@CXCL8@exo-miR-126a-3p on macrophage polarization via flow cytometry in over PIK3R2 macrophage (*n* = 3). All data were presented as mean ± SD. Compared with control group: ^#^*P* < 0.05, ^##^*P* < 0.01. Compared with LPS or over PIK3R2 group: *P < 0.05, **P < 0.01.

To verify that the effect of EGFR@CXCL8@exo-miR-126a-3p on macrophages was mediated by PIK3R2, this study used PIK3R2 plasmid to establish overexpressed PIK3R2 macrophages. The results showed the mRNA and protein expression of PIK3R2 was significantly increased in overexpressed PIK3R2 macrophages as compared with the control group (Fig. 10A to C). Further, the related proteins of NLRP3 signaling pathway and ferroptosis were evaluated. The results showed that EGFR@CXCL8@exo-miR-126a-3p significantly decreased the levels of NLRP3, ASC, GSDMD-N, cleaved caspase-1, and cleaved IL-1β and increased SLC7A11, GPX4, and NRF-2, and over PIK3R2 canceled the effects of EGFR@CXCL8@exo-miR-126a-3p on NLRP3 signaling pathway and ferroptosis (Fig. 10D and E). In addition, flow cytometry also showed over PIK3R2 the effects of EGFR@CXCL8@exo-miR-126a-3p on macrophage polarization (Fig. 10F and G).

## Discussion

ALI is one of the most severe and difficult to treat complications. However, effective clinical drugs have not yet been developed to treat ALI. In this study, EGFR and CXCL8 dual-responsive biomimetic particles (EGFR@CXCL8@exo-miR-126a-3p) were prepared to improve ALI.

In recent years, an increasing number of studies have suggested that EGFR was activated by LPS and played an important regulatory role in the LPS/TLR4 signaling pathway. The activated EGFR receptor significantly promoted the release of inflammatory factors such as TNF-α and IL-6 induced by LPS [20]. There were reports that knocking out EGFR in macrophages increased the production of pro-inflammatory cytokine TNF-α and anti-inflammatory cytokine IL-10 [21]. The above report stated that EGFR was one of the markers for macrophage activation and can serve as a target for macrophage response. IL-8, also known as chemokine CXCL8, is a cytokine secreted by macrophages and epithelial cells and can bind to its receptor CXCL8. CXCL8 was one of the important biomarkers for chemotactic targeting of macrophages. Therefore, this study utilized EGFR and CXCL8 to design EGFR@CXCL8@exo-loaded miR-126a-3p mimic for the treatment of ALI.

The bioinformatics software TargetScan was used to predict the target genes of miR-126a-3p. As both animal and cell models, we selected miR-126a-3p for target gene prediction. In addition, since the ultimate research objective was for clinical use, we need to select target genes that were shared for research. In this study, there were a total of 3 target genes shared, namely, PIK3R2, KANK2, and FBXO33. Finally, we chose PIK3R2 as the target gene for miR-126a-3p. PIK3R2 is a potential target gene for miR-126a-3p, which has been extensively studied in tumors [22,23] and has also been reported in myocardial hypertrophy and LPS-induced sepsis ALI [24,25]. In order to further explore the mechanism of effects of miR-126a-3p in ALI, this study first predicted the target of miR-126a-3p due to PIK3R2 through the software. The luciferase experiment confirmed the targeting relationship between miR-126a-3p and PIK3R2, that is, the targeted binding between miR-126a-3p and PIK3R2 occurred. In order to further verify that the effects of EGFR@CXCL8@exo-miR-126a-3p on ALI were achieved by targeting PIK3R2, PIK3R2 recovery experiments were used to overexpress PIK3R2 in macrophages. In addition, the results showed that overexpression of PIK3R2 could reverse the effects of EGFR@CXCL8@exo-miR-126a-3p on macrophage polarization and NLRP3 signaling pathway and ferroptosis, indicating that EGFR@CXCL8-exo-miR-126a-3p inhibited LPS-induced macrophage polarization and NLRP3 signaling pathway and ferroptosis by targeting PIK3R2 to alleviate ALI.

Ferroptosis is a novel cell death method discovered in recent years, which has been confirmed to be involved in organ damage in sepsis. When iron death worsens, intracellular iron accumulation causes an increase in toxic lipid peroxidation reactive oxygen species (ROS) and oxidative stress levels [26]. In this study, RNA-seq revealed that EGFR@CXCL8-exo-miR-126a-3p mainly intervenes in ALI by regulating SLC7A11. SLC7A11 is the main subunit responsible for the functioning of the SystemXc system. Its main function is to exchange extracellular cysteine and intracellular glutamate in a 1:1 ratio, providing sufficient cysteine to synthesize GSH. GSH can work together with GPX4 to convert intracellular lipid oxides into nontoxic lipid alcohols, reducing the accumulation of intracellular lipid oxides and inhibiting the occurrence and development of ferroptosis, thereby reducing tissue damage [27]. In this study, EGFR@CXCL8-exo-miR-126a-3p increased SLC7A11, GPX4, and NRF-2.

In conclusion, this study was the first innovatively constructed EGFR and CXCL8 dual-responsive biomimetic particle-loaded miR-126a-3p for the treatment of ALI via inhibition of PIK3R2/NLRP3 signaling pathway and ferroptosis. This study provides a novel strategy for the treatment of ALI.

## Ethical Approval

All procedures were approved by the Institutional Ethics Committee for Animal Experimentation of Nanjing Medical University (IACUC-2507088).

## Data Availability

The data underlying this article are available on reasonable request to the corresponding authors.
