# Supplementary material for: Double-Responsive Macrophage-Derived Exosomes Alleviate Acute Lung Injury
Source: Biomater Res. 2025 Nov 24;29:0277. doi: 10.34133/bmr.0277 (PMC12641162; doi:10.34133/bmr.0277)
Supplement: Supplementary 1 — Figs. S1 to S8 [file bmr.0277.f1.docx]

**Figure S1 Expression of EGFR and CXCL8 in macrophages**

**
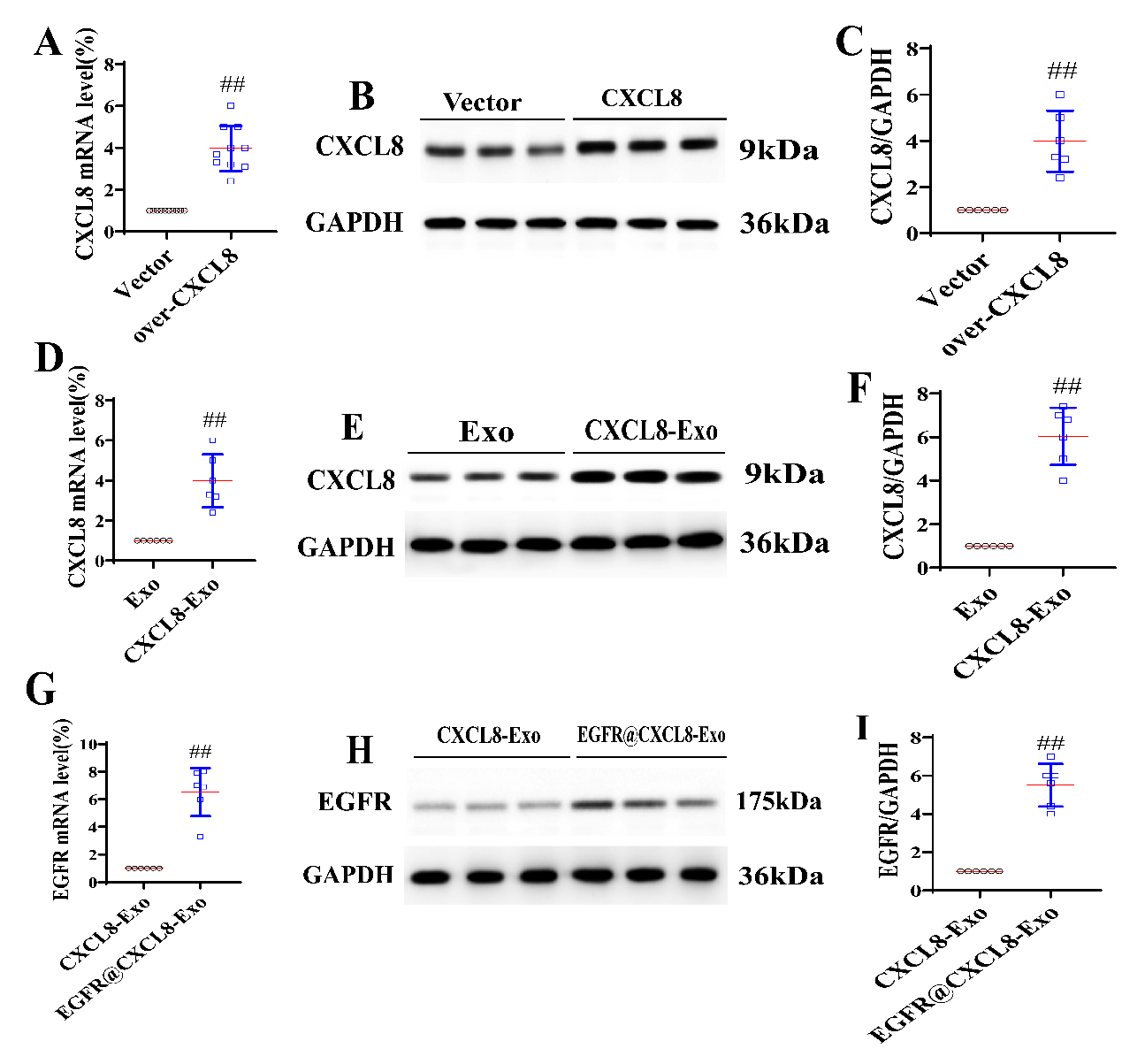
**

(A-C) The mRNA and proteins of CXCL8 in overexpressed CXCR8 macrophages (n=6); (D-F) The mRNA and proteins of CXCL8 in CXCR8-exo (n=6); (G-I) The mRNA and proteins of EGFR in EGFR@CXCL8-exo (n=6). All data were presented as mean ± SD. Compared with vector or CXCL8-exo group: ^#^P<0.05, ^##^P<0.01.

**Figure S2 The effect of NLRP3 downstream gene of PIK3R2 on cell pyroptosis**

**
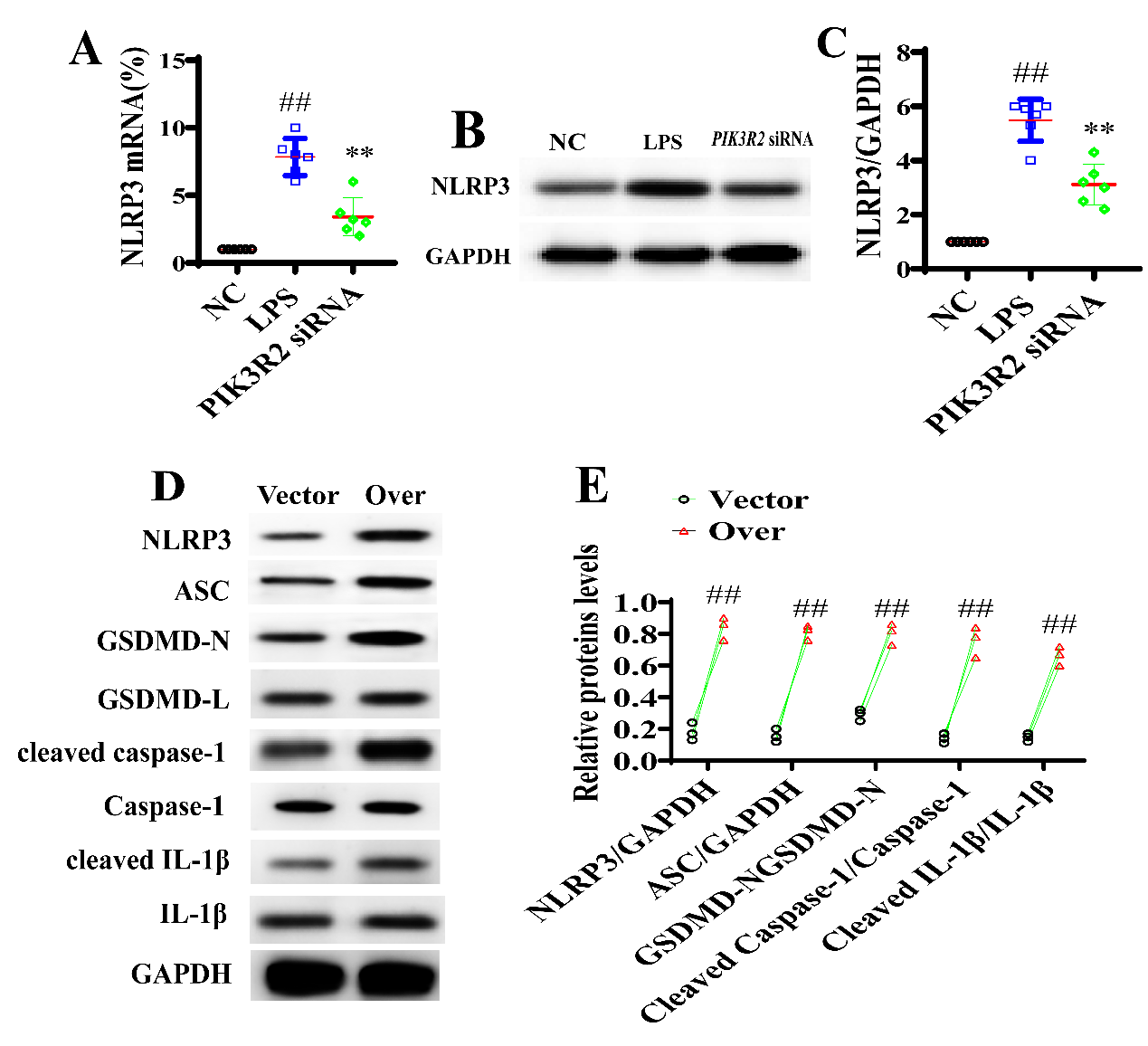
**

(A-C) The mRNA and proteins of NLRP3 in PIK3R2 siRNA macrophages (n=6); (D-E) The levels of NLRP3, ASC, cleaved Caspase-1 and cleaved-IL-1β (n=6). All data were presented as mean ± SD. Compared with vector group: ^#^P<0.05, ^##^P<0.01.

**Figure S3 RNA sequencing searches for EGFR@CXCL8-exo-miR-126a-3p signaling pathway**


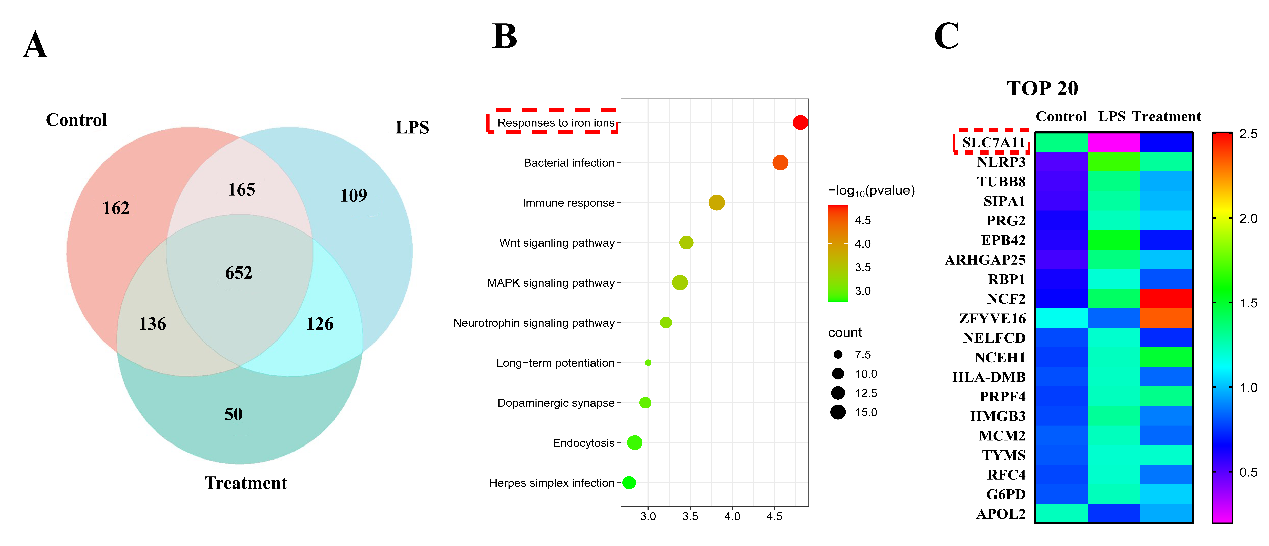


(A) Three gene enrichment Venn diagram (n=6); (B) Gene enrichment map (n=6); (C) Top 20 differentially expressed genes (n=6).

**Figure S4 The effects of macrophages on Treg cells**

**
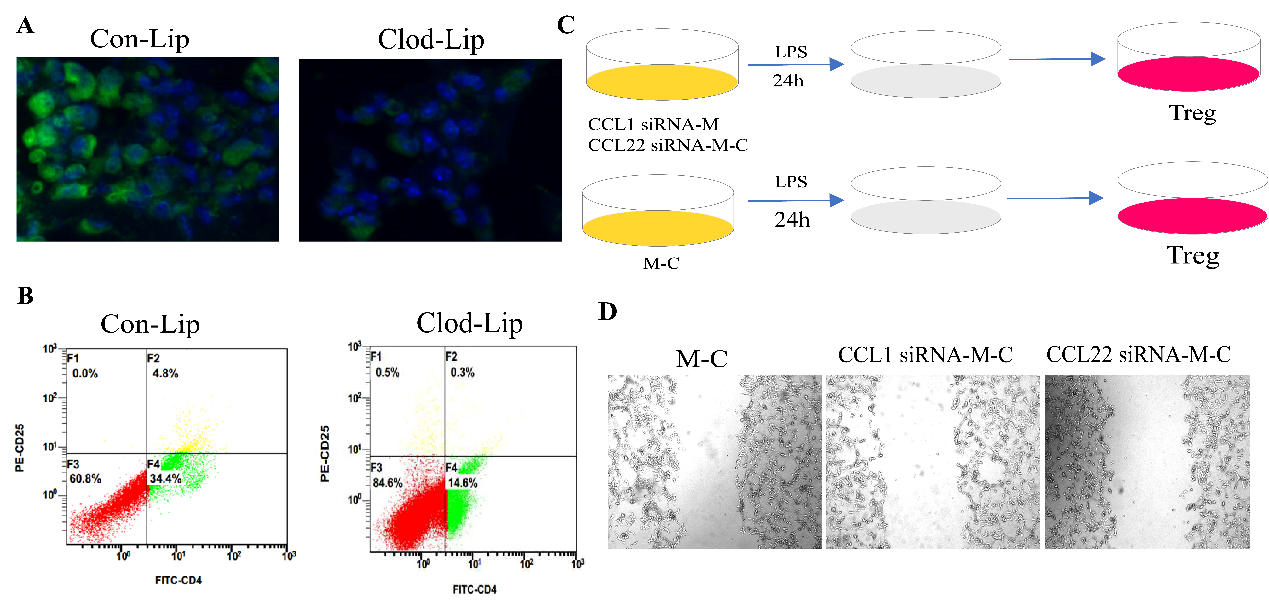
**

(A) Results of from mouse lung tissues (n=6); (B) The effects of macrophages cleared on Treg cell migration (n=6); (C) The effect of macrophage specific knockout of CCL1 and CCL22 on Treg cell migration(n=6).

**Figure S5 The effects of EGFR@CXCL8-exo-miR-126a-3p on w/d, MPO, pulmonary pathology and pulmonary function in ALI mice**

**
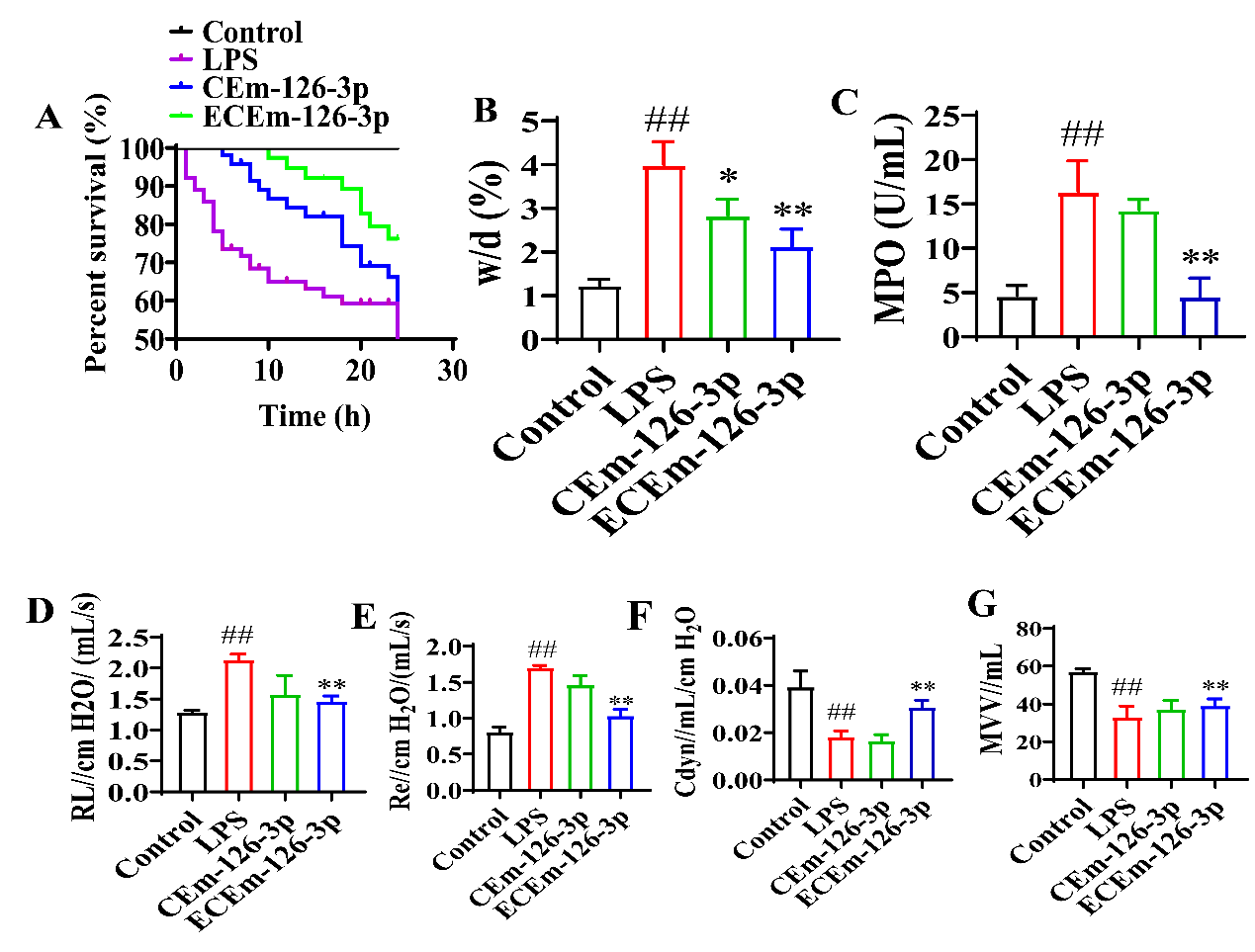
**

(A)Survival rate (n=6); (B)w/d (n=6); (C) MPO (n=6); (D-G) RL, Re, Cdyn and MVV(n=6). All data were presented as mean ± SD. Compared with control group: ^#^P<0.05, ^##^P<0.01; Compared with LPS group: ^*^P<0.05, ^**^P<0.01.

**Figure S6 The effects of EGFR@CXCL8-exo-miR-126a-3p on cytokine in serum, lung tissues and BALF**


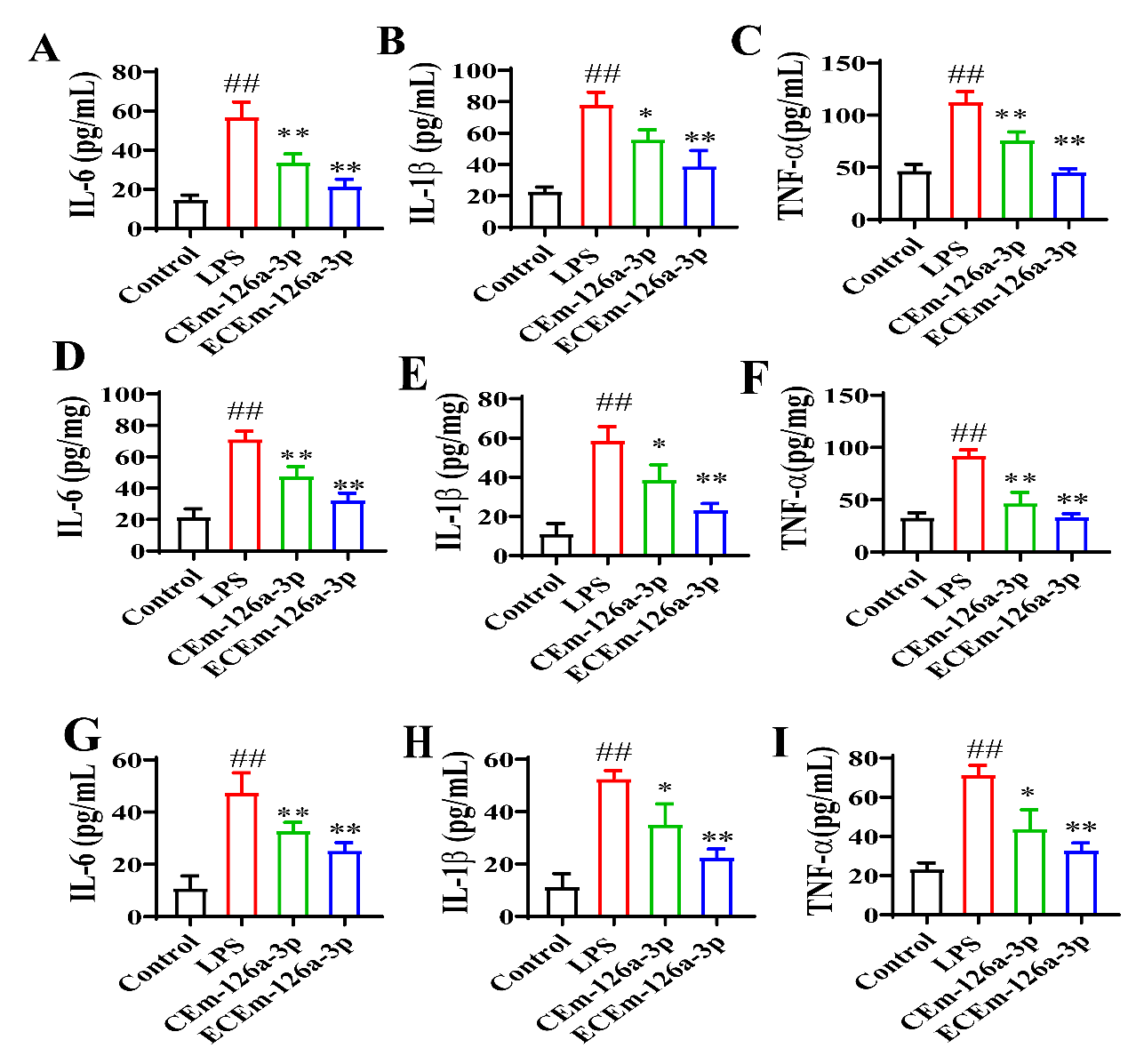


(A-C) The levels of TNF-α, IL-1β, IL-6 in serum (n=6); (B) The levels of TNF-α, IL-1β, IL-6 in lung tissues (n=6); (C) The levels of TNF-α, IL-1β, IL-6 in BALF (n=6). All data were presented as mean ± SD. Compared with control group: ^#^P<0.05, ^##^P<0.01; Compared with LPS group: ^*^P<0.05, ^**^P<0.01.

**Figure S7 The effects of EGFR@CXCL8-exo-miR-126a-3p on Tregs**


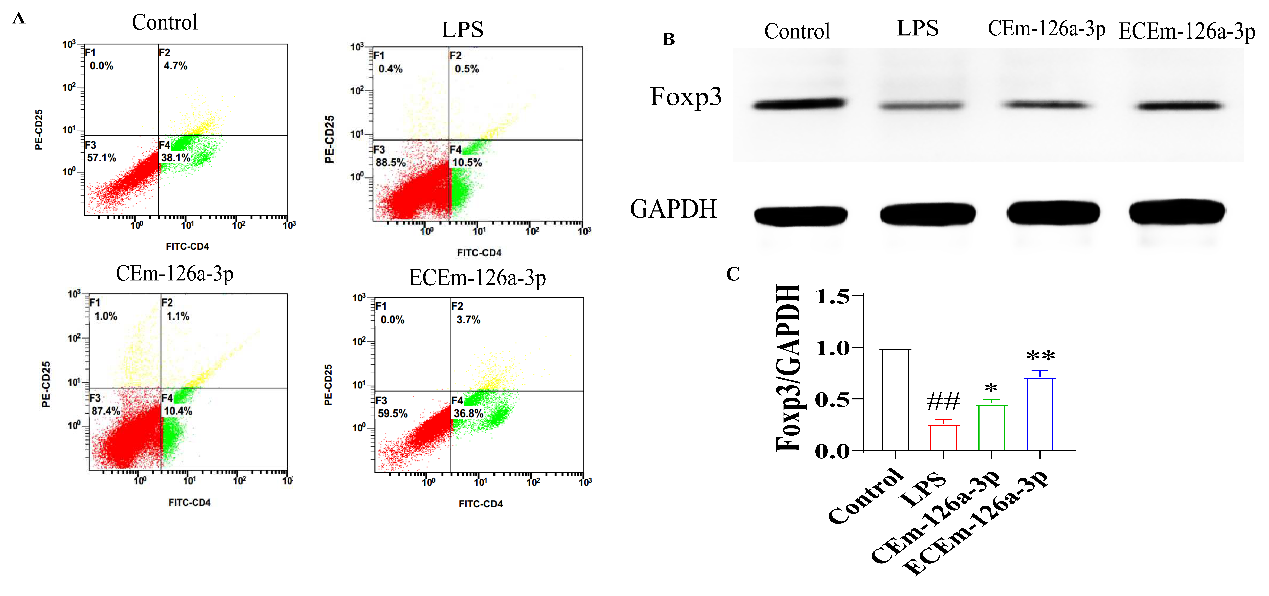


(A) Flow cytometry detection of Tregs in lung tissues(n=6); (B-C) The levels of Foxp3 (n=6). All data were presented as mean ± SD. Compared with control group: ^#^P<0.05, ^##^P<0.01; Compared with LPS group: ^*^P<0.05, ^**^P<0.01.

**Figure S8 The effects of EGFR@CXCL8-exo-miR-126a-3p on cytokine in cell supernatant**

**
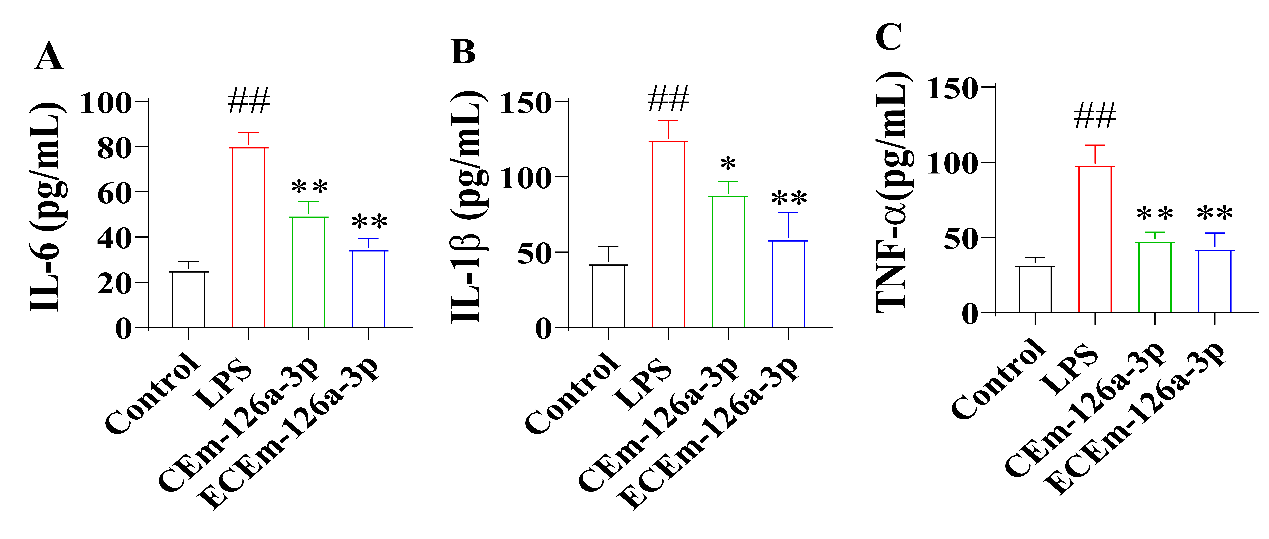
**

(A-C) The levels of TNF-α, IL-1β, IL-6 in serum (n=6). All data were presented as mean ± SD. Compared with control group: ^#^P<0.05, ^##^P<0.01; Compared with LPS group: ^*^P<0.05, ^**^P<0.01.
